# Revision of the genus *Hydrobiomorpha* Blackburn, 1888 (Coleoptera, Hydrophilidae) from Argentina, with the description of a new species and an updated identification key

**DOI:** 10.3897/zookeys.1289.202453

**Published:** 2026-08-11

**Authors:** Matías R. Urcola, Georgina Rodriguez, Mariano C. Michat, Patricia L.M. Torres

**Affiliations:** 1 Universidad de Buenos Aires, Facultad de Ciencias Exactas y Naturales, Departamento Biodiversidad y Biología Experimental, Laboratorio de Entomología, Buenos Aires, Argentina Universidad de Buenos Aires, Facultad de Ciencias Exactas y Naturales, Departamento Biodiversidad y Biología Experimental, Laboratorio de Entomología Buenos Aires Argentina; 2 CONICET-UBA, Instituto de Biodiversidad y Biología Experimental y Aplicada (IBBEA), Buenos Aires, Argentina CONICET-UBA, Instituto de Biodiversidad y Biología Experimental y Aplicada (IBBEA) Buenos Aires Argentina

**Keywords:** Anatomy, Hydrophilini, Neotropical Region, new species, water scavenger beetles

## Abstract

The genus *Hydrobiomorpha* Blackburn, 1888 is a pantropical group of aquatic beetles that play important roles in freshwater ecosystems as predators (larvae) and scavengers (adults). In South America, 31 species are known, with six previously recorded from Argentina: *Hydrobiomorpha corumbaensis* Mouchamps, 1959, *H. iguazu* Bachmann, 1988, *H. irina irina* (Brullé, 1837), *H. longa* (Bruch, 1915), *H. rudesculpta* (Orchymont, 1939) and *H. spinosa* (Orchymont, 1928). Building on the foundational contributions of Dr. Axel O. Bachmann to the taxonomy of the genus in the Americas, a comprehensive revision of the Argentine species of *Hydrobiomorpha* is presented, integrating high-resolution digital photography and scanning electron microscopy to document anatomical features in detail. Seven species are recognized, including *H. bachmanni***sp. nov**., described from Argentina (Corrientes Province) and Paraguay, dedicated to Dr. Bachmann in recognition of his legacy. For comparative purposes, *H. utiariti* Bachmann, 1988 is also treated, given its anatomical similarity to the new species. An updated identification key to the Argentinean species is provided. The presence, extent, and location of the male mesotibial setigerous furrow, claw structure, the glabrous area on the last abdominal ventrite, and male genitalia are evaluated as key diagnostic characters, revealing consistent interspecific variation. Distribution patterns show highest species richness in northeastern Argentina, with *H. spinosa* (Orchymont, 1928) exhibiting the broadest range and *H. iguazu* Bachmann, 1988 restricted to Misiones Province. This revision provides a modern, visually documented taxonomic framework that facilitates accurate species identification for future phylogenetic, ecological, and conservation studies.

## Introduction

The genus *Hydrobiomorpha* Blackburn, 1888 (Coleoptera: Hydrophilidae) comprises medium to large-sized aquatic beetles (13.7–28.0 mm in body length) belonging to the subtribe Hydrophilina, whose monophyly is supported by a modified aedeagus with a distinctive laterally compressed median lobe and strongly prolonged lateral margins of the basal piece (Short 2010). The genus has a predominantly tropical distribution encompassing the Neotropical, Afrotropical, and Indo-Australian regions, extending into the southernmost part of the Nearctic. Currently, the genus includes 61 valid species (56 extant and five fossil) and 15 subspecies (Newton 2022). Within the Neotropical region, 31 species are recorded for South America, six of which were reported from Argentina (Bachmann 1988). Aquatic Hydrophilidae, including *Hydrobiomorpha*, play important roles in freshwater ecosystems as predators (larvae) and scavengers (adults), and are increasingly recognized as valuable bioindicators of aquatic diversity and environmental quality (Bilton et al. 2019; Archangelsky et al. 2023). However, the alpha taxonomy of many groups remains obscure, limiting their ecological and conservation studies.

Historically, the knowledge of *Hydrobiomorpha* in Argentina has been based on the work of Dr. Axel O. Bachmann. He described numerous species, clarified synonymies, and established the taxonomic and faunistic foundations for the study of this group at national and regional levels (Bachmann 1963, 1988). The diagnostic characters and the species themselves were originally described and illustrated using line drawings and text-based descriptions. Additionally, the identification keys available for the region (Oliva et al. 2002) have not been updated to reflect current species diversity or to incorporate modern imaging techniques.

In this study, we present a comprehensive taxonomic revision of the Argentine species of *Hydrobiomorpha*, integrating high-resolution digital photography and scanning electron microscopy to document diagnostic anatomical features in detail. We provide revised diagnoses for all known Argentinean species, describe a new species dedicated to Dr. Axel O. Bachmann in recognition of his contributions to the taxonomy of *Hydrobiomorpha* in the Americas, and present an updated identification key to all Argentine species.

## Materials and methods

The examined material is deposited in the collections of the Museo Argentino de Ciencias Naturales “Bernardino Rivadavia” (**MACN**) and the Laboratory of Entomology, Facultad de Ciencias Exactas y Naturales, Universidad de Buenos Aires (**LEBA**), Ciudad Autónoma de Buenos Aires, Argentina. In this study, we also include *Hydrobiomorpha utiariti* Bachmann, 1988, a species originally described from Brazil and Paraguay. This species is incorporated because its male genitalia shows a close resemblance to those of the new species described herein, and because it occurs in Paraguay, a country where the new species is also found. Specimens that included only collection site information were georeferenced using Google Earth (Google LLC, Mountain View, CA, USA), providing the latitude and longitude within square brackets, which represent locality coordinates estimates rather than the precise collection site.

Type labels are transcribed verbatim as indicated by quotation marks. Forward slashes (/) indicate line breaks on the respective label.

Specimens were studied using a Leica MZ6 stereomicroscope. Measurements of the largest and smallest individuals—length from the clypeal apex to the elytral apex (**L**) and maximum width (**W**)—were taken with an ocular micrometer.

Male genitalia from paratype specimens were dissected and cleared in lactic acid for examination. For the description of the holotype of the new species, anatomical features were documented using figures, some of which were derived from paratype specimens. Prior to this, we verified that the relevant characters were constant between the holotype and the paratypes. This approach was adopted to avoid dissecting or otherwise damaging the holotype, thereby preserving the integrity of the primary type specimen. For photographic documentation, a Nikon D800e digital camera coupled to a macro lens system was used. Habitus images were captured using a Nikon AF-S VR Micro-Nikkor 105 mm f/2.8G IF-ED lens with a Raynox DCR-250 (Tokyo, Japan) auxiliary lens. Details of the male genitalia and the glabrous area of the fifth abdominal ventrite were photographed by replacing the auxiliary lens with a Raynox MSN-202 (Tokyo, Japan). Claws were photographed using the Raynox MSN-505 auxiliary lens (Tokyo, Japan).

All photographs were taken as focal stacks, which were merged digitally using Helicon Focus 6.7.1 Pro (Kharkiv, Ukraine) and finalized (level adjustments, cropping, background) with Adobe Photoshop CC 2019. Dorsal, lateral, and ventral habitus photographs of all *Hydrobiomorpha* species treated in this paper are available on Zenodo at https://doi.org/10.5281/zenodo.21263839.

Selected anatomical structures—head, pronotum, elytron and the setigerous furrow on the ventral (inner) face of the male mesotibiae— were documented using scanning electron microscopy (SEM). Specimens for SEM were prepared following a standard protocol: superficial cleaning with a soft brush, sonication for 4 min in warm water with detergent and again for 4 min in a commercial glass cleaner, dehydration in a graded ethanol series, air-drying overnight at room temperature, mounting on stubs with copper tape, and sputter-coating with gold-palladium. SEM images were acquired with a Zeiss GeminiSEM 360 microscope at the MACN.

The holotype ♂ and paratypes of *H. bachmanni* sp. nov. are deposited in the entomological collection of the Museo Argentino de Ciencias Naturales “Bernardino Rivadavia” (**MACN**), Ciudad Autónoma de Buenos Aires, Argentina.

## Taxonomy

### Family Hydrophilidae Latreille, 1802

**Tribe Hydrophilini Latreille, 1802**

#### Genus Hydrobiomorpha

Taxon classificationAnimaliaColeopteraHydrophilidae

Blackburn, 1888

E6B43B05-0A8B-59D5-AADB-92B7BE4C0892

Hydrobiomorpha Blackburn, 1888: 814–815. (Type species: Hydrobiomorpha bovilli Blackburn, 1889, by subsequent designation of Knisch (1924: 234).
Neohydrophilus d’Orchymont, 1911: 59. (Type species: Neohydrophilus deplanatus, d’Orchymont, 1919, by original designation). Originally described as a subgenus of Hydrophilus; raised to generic rank by d’Orchymont 1919: 160–161; synonymized with Hydrobiomorpha by Mouchamps 1959: 296.

##### Taxonomic history.

Blackburn (1888) established the genus *Hydrobiomorpha* for two Australian species, *H. bovilli* and *H. tepperi*, but no type species was designated. Later, Knisch (1924) designated *H. bovilli* Blackburn as the type species. *Hydrobiomorpha bovilli* is a poorly known species and its type material has not been examined by recent authors. Bachmann (1988) examined specimens of *H. tepperi*, considered closely similar to *H. bovilli* by Blackburn (1888) and Mouchamps (1959), and concluded that the placement of *H. bovilli* in *Hydrobiomorpha* is justified. d’Orchymont (1911) established the subgenus Hydrophilus (Neohydrophilus) for several species, without designating a type species. In 1919, he raised *Neohydrophilus* to generic rank and designated *N. deplanatus* as the type species. Mouchamps (1959) revised the group, synonymized *Neohydrophilus* with *Hydrobiomorpha*, and added several new species, four from the Americas. Bachmann (1988) revised the American members of the group and added 19 new species. The genus currently comprises 61 species, 56 of which are extant and have a pantropical distribution (Hansen 1999; Short and Hebauer 2006; Short 2010; Short and Fikáček 2011; Newton 2022).

##### Diagnosis.

Medium-sized to large beetles, length 13.7–28.0 mm (most species 17.0–22.0 mm); body moderately to strongly convex in lateral view; color uniformly black, or more frequently dark brown, without metallic sheen or paler areas; legs slightly paler, rarely yellowish brown (only some *H. rudesculpta*); clypeus excavate anteriorly, separated from labrum by membranous stripe; labrum with two coarse setigerous punctures; genae near gula partly glabrous; several antennomeres (including club) moderately expanded, divided, with long setae; prosternum, forming a median keel, ventral border generally straight and subparallel to body axis, sometimes rounded, oblique or inflexed, and most frequently with posterior sharp point, rarely very short; meso-metasternal keel straight or slightly concave in lateral view, with small anterior notch; posterior portion short, rarely surpassing posterior margin of first abdominal ventrite, ending bluntly and laterally compressed, sometimes subconical and sharply pointed, or slightly hooked; furcasternum pubescent; mesotibiae of males sometimes with setigerous furrow along distal half of ventral (inner) face, of variable length, covered with dense brush of golden to reddish setae; claws falciform, with proximal denticle, claws of same leg frequently differing slightly in size or shape; male claws strongly angulate; elytra without sutural stria; marginal series of setigerous punctures very regular, sometimes irregular, rarely indistinct; abdominal ventrites totally covered by hydrophobic pubescence, except for fifth ventrite, which shows semicircular, subrectangular or subtriangular posterior glabrous area, typically larger in males, rarely minute, or absent in females; male genitalia complex, median lobe with dorsal sclerite large, variously shaped, laterally compressed, and frequently with raised dorsal lamina; gonopore located proximally or at mid-length, with conspicuous ventral sclerite (“ventral strut” of Leech in Bachmann 1988); accessory appendages of median lobe variable in shape, ranging from broad to elongate and from straight to curved or sigmoid, with apices acute, truncate, rounded, or bearing short outwardly directed hooks; parameres bent, with accessory appendages robust, sclerotized, rarely at right angle, or largely bifid.

##### Distribution.

Pantropical, mainly Neotropical. Contartese and Bachmann (1987) presented maps with the distribution of the species of Argentina.

##### Biology.

Adults and larvae inhabit vegetated permanent ponds but are infrequently collected; to date, no records from rain pools are known. Adults are mostly scavengers or periphyton eaters, and larvae are predaceous (Archangelsky et al. 2004). Adults are good swimmers, occasionally flying at night and attracted to artificial lights.

#### Hydrobiomorpha bachmanni sp. nov.

Taxon classificationAnimaliaColeopteraHydrophilidae

50AE4B68-84D6-5937-A484-5E1B2DDE2230

https://zoobank.org/2A3BD11A-4AE4-4A6B-933A-2479C168B79C

Figs 1A, 2A, 3A, 4A, 4B, 5A–D, 6A–L, 7A, 7G, 8

##### Type locality.

Argentina: Corrientes Province, Parque Nacional Iberá, Portal Laguna Iberá, 28°32'46"S, 57°11'45"W, elevation 67 m a.s.l.

##### Type material.

***Holotype*** • ♂, deposited in the entomological collection of the Museo Argentino de Ciencias Naturales “Bernardino Rivadavia” (MACN), Buenos Aires, Argentina, labelled: “ARGENTINA. Corrientes / Province. PN Iberá. Portal / Laguna Iberá 8.xi.2019 / JI Urcola and G Rodriguez leg.” [typed, white label], “HOLOTYPE / *Hydrobiomorpha bachmanni* sp. nov. / Urcola, Rodriguez / Michat, Torres, 2026" [typed, red label], “MACN_En / 46706" [typed, white label]. ***Paratypes***: • 10 ♂♂ and 10 ♀♀ from type locality (MACN_En 46707 to 46726). All paratypes with the following labels: “ARGENTINA. Corrientes / Province. PN Iberá. Portal / Laguna Iberá 14.xi.2019 / JI Urcola and G Rodriguez leg.” [typed, white label], “PARATYPE / *Hydrobiomorpha bachmanni* sp. nov. / Urcola, Rodriguez / Michat, Torres, 2026" [typed, yellow label].

##### Other material examined.

Paraguay: • Presidente Hayes Department, Estancia Campo Grande, 24°23'07"S, 57°19'21"W, 5–8.xii.2002, OR Di Iorio leg., light trap, 6 ♂♂ and 5 ♀♀ (LEBA).

##### Diagnosis.

**Figure 1.**
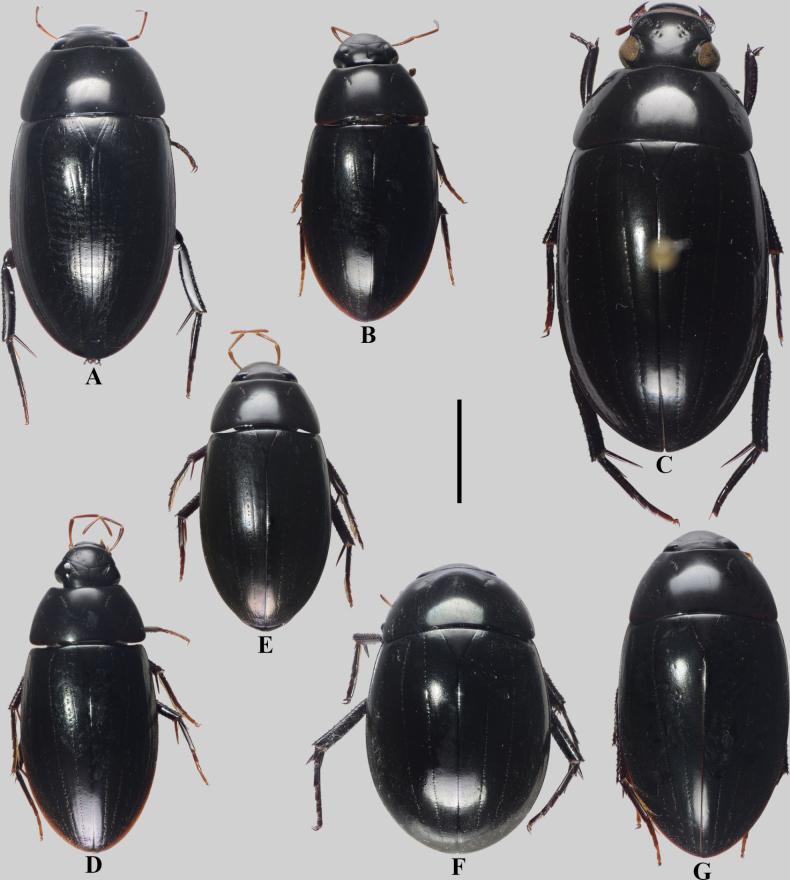
Habitus of *Hydrobiomorpha* species, dorsal aspect. **A**. *Hydrobiomorpha bachmanni* sp. nov. (holotype, MACN-En 46706); **B**. *Hydrobiomorpha corumbaensis*; **C**. *Hydrobiomorpha iguazu* (holotype, MACN-En 2213); **D**. *Hydrobiomorpha irina irina*; **E**. *Hydrobiomorpha longa*; **F**. *Hydrobiomorpha rudesculpta*; **G**. *Hydrobiomorpha spinosa*. Scale bar: 5 mm.

**Figure 2.**
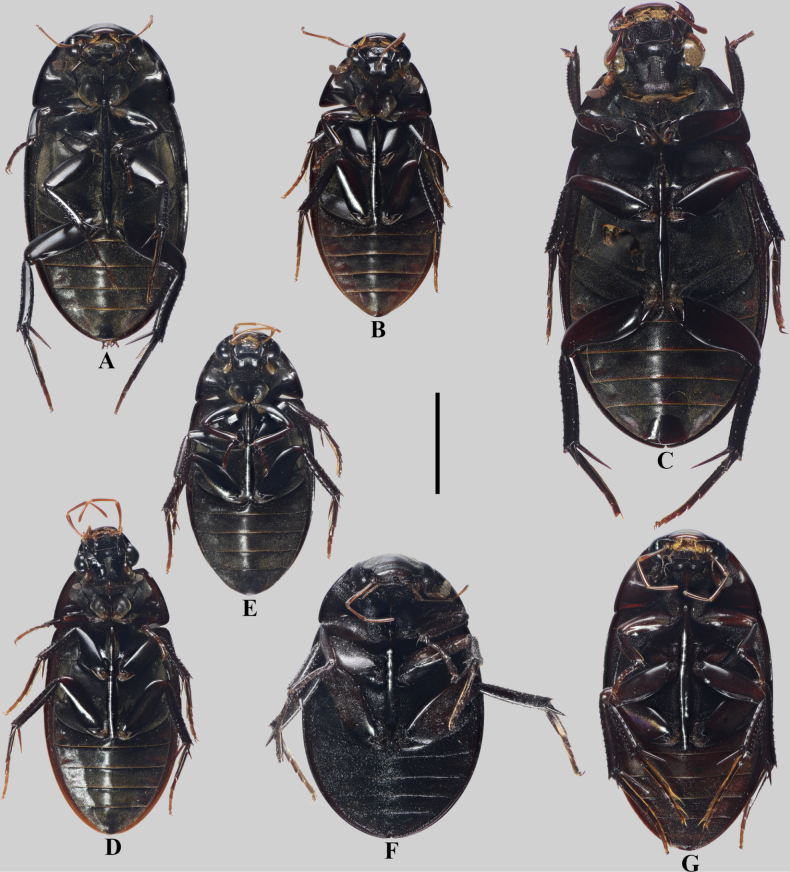
Habitus of *Hydrobiomorpha* species, ventral aspect. **A**. *Hydrobiomorpha bachmanni* sp. nov. (holotype, MACN-En 46706); **B**. *Hydrobiomorpha corumbaensis*; **C**. *Hydrobiomorpha iguazu* (holotype, MACN-En 2213); **D**. *Hydrobiomorpha irina irina*; **E**. *Hydrobiomorpha longa*; **F**. *Hydrobiomorpha rudesculpta*; **G**. *Hydrobiomorpha spinosa*. Scale bar: 5 mm.

**Figure 3.**
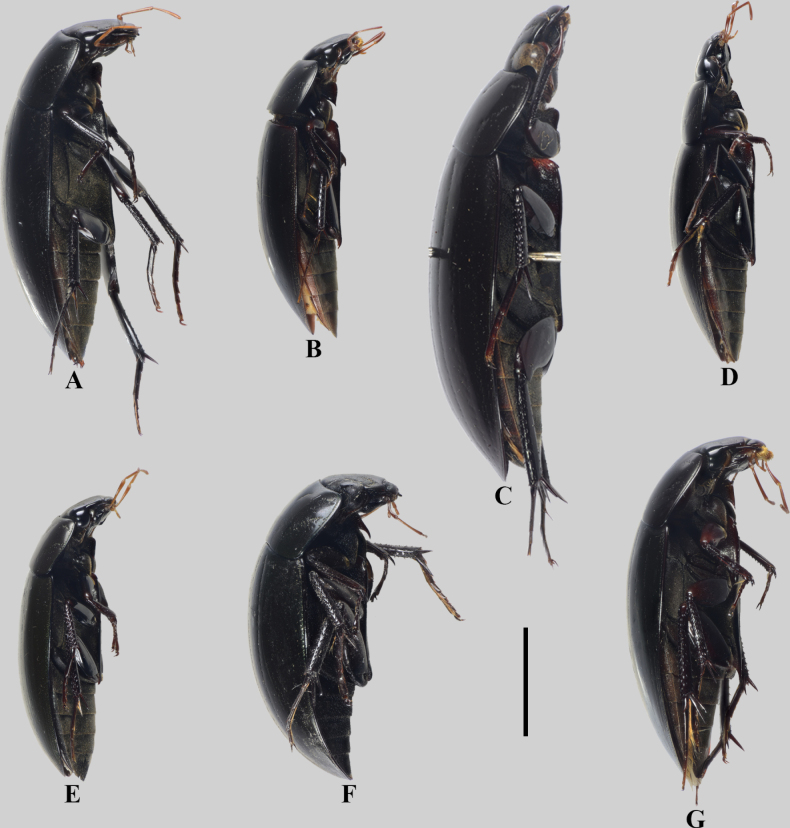
Habitus of *Hydrobiomorpha* species, lateral aspect. **A**. *Hydrobiomorpha bachmanni* sp. nov. (holotype, MACN-En 46706); **B**. *Hydrobiomorpha corumbaensis*; **C**. *Hydrobiomorpha iguazu* (holotype, MACN-En 2213); **D**. *Hydrobiomorpha irina irina*; **E**. *Hydrobiomorpha longa*; **F**. *Hydrobiomorpha rudesculpta*; **G**. *Hydrobiomorpha spinosa*. Scale bar: 5 mm.

**Figure 4.**
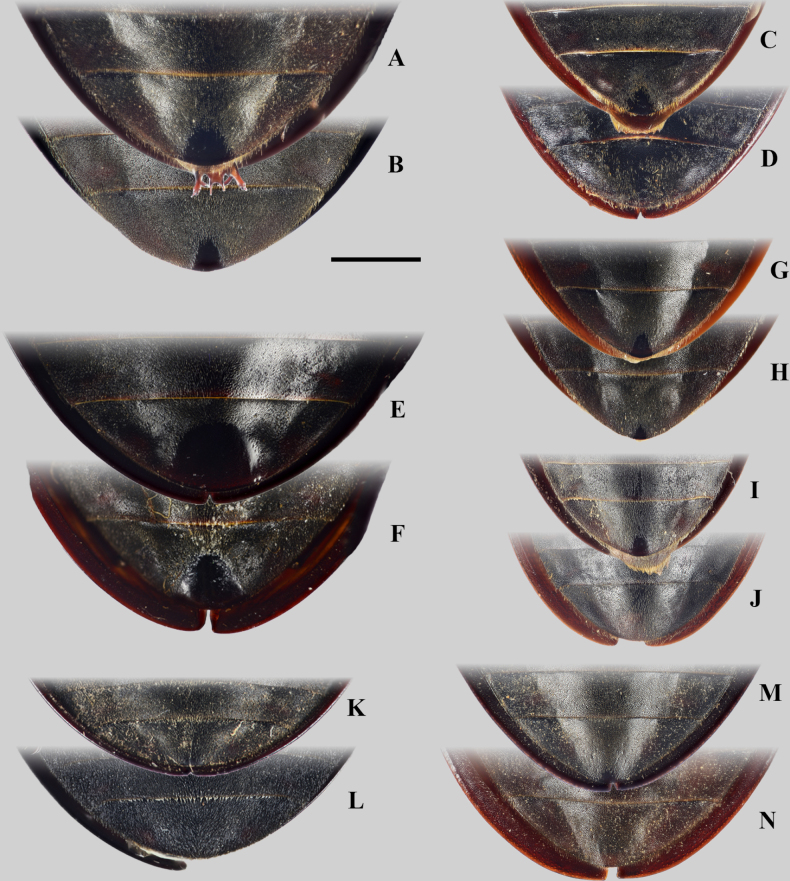
Abdominal ventrites IV–V of *Hydrobiomorpha* species, ventral aspect. **A, B**. *Hydrobiomorpha bachmanni* sp. nov.; **C, D**. *Hydrobiomorpha corumbaensis*; **E, F**. *Hydrobiomorpha iguazu*; **G, H**. *Hydrobiomorpha irina irina*; **I, J**. *Hydrobiomorpha longa*; **K, L**. *Hydrobiomorpha rudesculpta*; **M, N**. *Hydrobiomorpha spinosa*. **A, C, E, G, I, K, M**. Males; **B, D, F, H, J, L, N**. Females. Scale bar: 2 mm.

**Figure 5.**
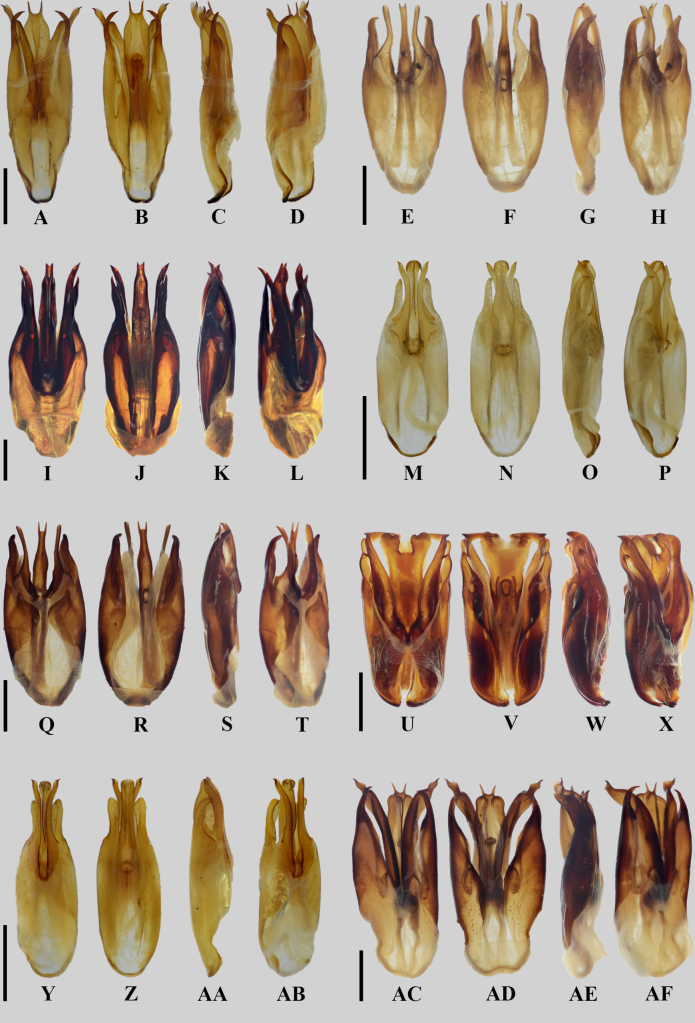
Male genitalia of *Hydrobiomorpha* species. **A–D**. *Hydrobiomorpha bachmanni* sp. nov. (paratype, MACN-En 46707); **E–H**. *Hydrobiomorpha corumbaensis*; **I–L**. *Hydrobiomorpha iguazu* (holotype, MACN-En 2213); **M–P**. *Hydrobiomorpha irina irina*; **Q–T**. *Hydrobiomorpha longa*; **U–X**. *Hydrobiomorpha rudesculpta*; **Y–AB**. *Hydrobiomorpha spinosa*; **AC–AF**. *Hydrobiomorpha utiariti* (holotype, MACN-En 2746). **A, E, I, M, Q, U, Y, AC**. Dorsal aspect; **B, F, J, N, R, V, Z, AD**. Ventral aspect; **C, G, K, O, S, W, AA, AE**. Right lateral aspect; **D, H, L, P, T, X, AB, AF**. Dorsal right-oblique aspect. Scale bars: 1 mm.

L: 15.5–19.0 mm (males), 16.5–20.5 mm (females). W: 7.5–8.5 mm (males), 8.0–9.4 mm (females). Body shape (Figs 1A, 2A, 3A): elliptical, sides subparallel, rather convex; elytral margins scarcely expanded outward on the anterior half. Prosternal keel (Fig. 3A) slightly concave; posterior spine acute, moderately long, reaching approximately mid-length of procoxae. Metaventral keel (Fig. 3A) almost straight in lateral view, with small anterior notch and midline depression; posterior portion short, weakly arched ventrally, apex obtusely pointed, not reaching first abdominal ventrite. Male: mesotibial setigerous furrow present, extending for ~ 1/3 of tibial length (Table 1; Fig. 7A, G); protarsal claws robust, anterior claw with a pointed proximal tooth; meso- and metatarsal claws with a conspicuous blunter proximal tooth; fifth abdominal ventrite with glabrous area occupying ~ 2/5 of its total length (Fig. 4A); genitalia: median lobe broad, apex deeply bifurcate into two long, narrow, subparallel projections; accessory appendages of median lobe broad, sclerotized, abruptly narrowed distally and terminating in short, apical hooks directed outward; parameres moderately broad in lateral view, apex acute and strongly sclerotized, curved toward midline (Fig. 5A–D). Female: fifth abdominal ventrite with glabrous area ~ 2/5 of its total length, though narrower than in males (Fig. 4B).

**Table 1.** Diagnostic characters separating species of *Hydrobiomorpha*. Fractions given for the mesotibial setigerous furrow indicate its relative length with respect to total mesotibial length; proportions of the glabrous area of the last abdominal ventrite correspond to the relative length of the glabrous patch with respect to the total length of the fifth abdominal ventrite.

**Species**	**L (in mm)**	**Mesotibial furrow (**♂)	**Glabrous area of last abdominal ventrite** ♂	**Glabrous area of last abdominal ventrite** ♀	**Prosternal keel (shape and extension)**	**Metaventral keel (extent)**	**Modification of male claws**
*H. bachmanni* sp. nov.	♂: 15.5–19.0	present (~1/3)	~2/5	~2/5	slightly concave; short	short, not reaching first abdominal ventrite	claws robust, proximal tooth pointed/blunt
	♀: 16.5–20.5						
*H. corumbaensis*	♂: 15.5–17.5	absent	~1/4–1/3	absent	nearly straight; short	short, not reaching first abdominal ventrite	claws dissimilar; posterior metatarsal claw expanded
	♀: 15.5–18.6						
*H. iguazu*	♂: 22.6	present (~1/4)	~3/4	~2/3	nearly straight; moderately long	long, reaching mid-length of first abdominal ventrite	claws robust, proximal tooth conspicuous
	♀: 21.0						
*H. irina irina*	♂: 14.2–16.0	absent	<1/4	≤1/5	nearly straight; short	very long, reaching or slightly surpassing posterior margin of first abdominal ventrite	claws slender, proximal tooth long and hooked
	♀: 14.5–17.2						
*H. longa*	♂: 14.0–15.3	absent	~1/3	absent	nearly straight; moderately long	short, not reaching first abdominal ventrite	claws modified; metatarsal claws angularly expanded
	♀: 14.8–16.6						
*H. rudesculpta*	♂: 16.3–18.8	absent	very short, broad	absent	strongly oblique; long	moderately long, not reaching mid-length of first abdominal ventrite	claws strongly modified, proximal tooth enormous
	♀: 17.2–20.3						
*H. spinosa*	♂: 13.7–17.9	present (~ 1/2 to 2/3)	<1/5 (often ~1/10)	absent	variable; generally long	very long, reaching or surpassing posterior margin of first abdominal ventrite	claws elongate, proximal tooth long and blunt
	♀: 14.0–20.5						
*H. utiariti*	♂: 22.0	present (~1/2)	~2/3	<1/2	straight; short	long, reaching mid-length of first abdominal ventrite	claws robust, proximal tooth spatulate
	♀: 21.0–22.5						

##### Differential diagnosis.

*Hydrobiomorpha bachmanni* sp. nov. can be readily distinguished from the other Argentine *Hydrobiomorpha* species by the unique combination of a glabrous area present in both sexes and occupying ~ 2/5 of the length of the last abdominal ventrite (Fig. 4A, B), together with a male mesotibial setigerous furrow extending along about the distal 1/3 of the tibia (Fig. 7A, G). In *H. iguazu*, the glabrous area of the last abdominal ventrite is much larger in both sexes (Fig. 4E, F) and the male mesotibial setigerous furrow extending along about the distal 1/4 of the tibia; in *H. spinosa*, the male mesotibial setigerous furrow extending along about the distal 1/2 of the tibia (Fig. 7F, J) and the glabrous area of the last abdominal ventrite is minute in male and absent in female (Fig. 4M, N); in *H. irina irina*, the glabrous area of last abdominal ventrite is much smaller in both sexes (Fig. 4G, H) and males lack the mesotibial furrow. The species also differs from *H. corumbaensis* and *H. longa*, both of which lack a glabrous area on the last abdominal ventrite in females (Fig. 4D, J) and a mesotibial furrow in males (Fig. 7B, D, H). The male genitalia of *H. bachmanni* (Fig. 5A–D) is readily distinguished from those of all other Argentine species by the median lobe with apex deeply bifurcate into two long, narrow, subparallel projections, and by the parameres with acute, strongly sclerotized apices and broad accessory appendages ending in short outwardly directed hooks. The most similar male genitalia are found in *H. utiariti* (Fig. 5AC–AF), but that species differs in having the median lobe apically truncate, with two shorter, slightly divergent projections, and parameres that are more strongly curved and denticulate subdistally.

**Figure 6.**
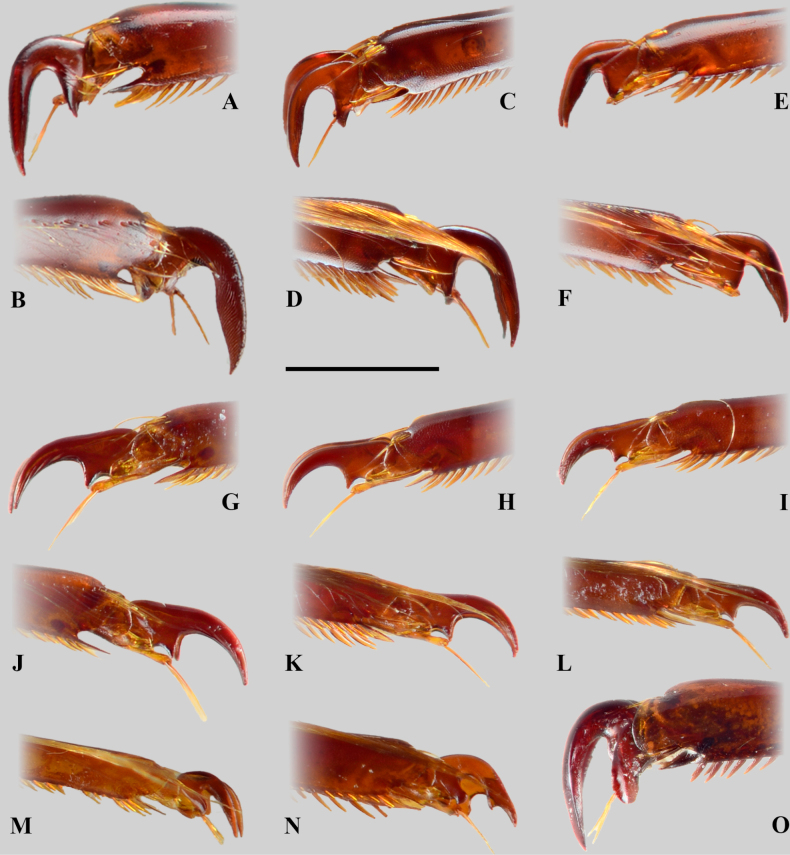
Claws of *Hydrobiomorpha* species. **A–L**. *Hydrobiomorpha bachmanni* sp. nov.; **M**. *Hydrobiomorpha corumbaensis*; **N**. *Hydrobiomorpha longa*; **O**. *Hydrobiomorpha rudesculpta*. **A, G, O**. Anterior claws of prothoracic leg; **B, J**. Posterior claws of prothoracic leg; **C, H**. Anterior claws of mesothoracic leg; **D, K**. Posterior claws of mesothoracic leg; **E, I**. Anterior claws of metathoracic leg; **F, L, M, N**. Posterior claws of metathoracic leg; **A–F, M–O**. Male specimens; **G–L**. Female specimen. Scale bar: 500 µm.

**Figure 7.**
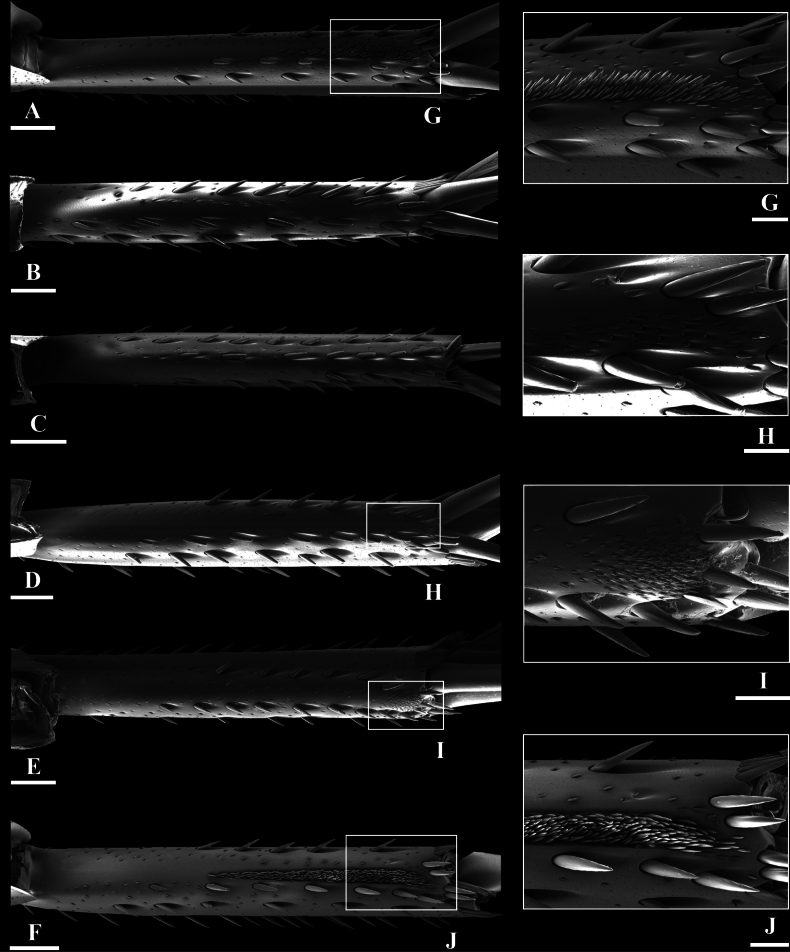
Mesotibiae of *Hydrobiomorpha* species. **A, G**. *Hydrobiomorpha bachmanni* sp. nov.; **B**. *Hydrobiomorpha corumbaensis*; **C**. *Hydrobiomorpha irina irina*; **D, H**. *Hydrobiomorpha longa*; **E, I**. *Hydrobiomorpha rudesculpta*; **F, J**. *Hydrobiomorpha spinosa*. **A–F**. Ventral (inner) views; **G–J**. Details of distal portion. Scale bars: 300 µm (**A–F**), 100 µm (**G, I–J**), 60 µm (**H**).

##### Description of holotype.

***Size***. L: 16.8 mm; W: 8.0 mm.

***Habitus***. Body elliptical, sides subparallel, rather convex.

***Color***. Integument very dark brown to black; venter slightly paler. Legs dark brown to black, with tarsi paler. Antennae and palpi light reddish.

**Figure 8.**
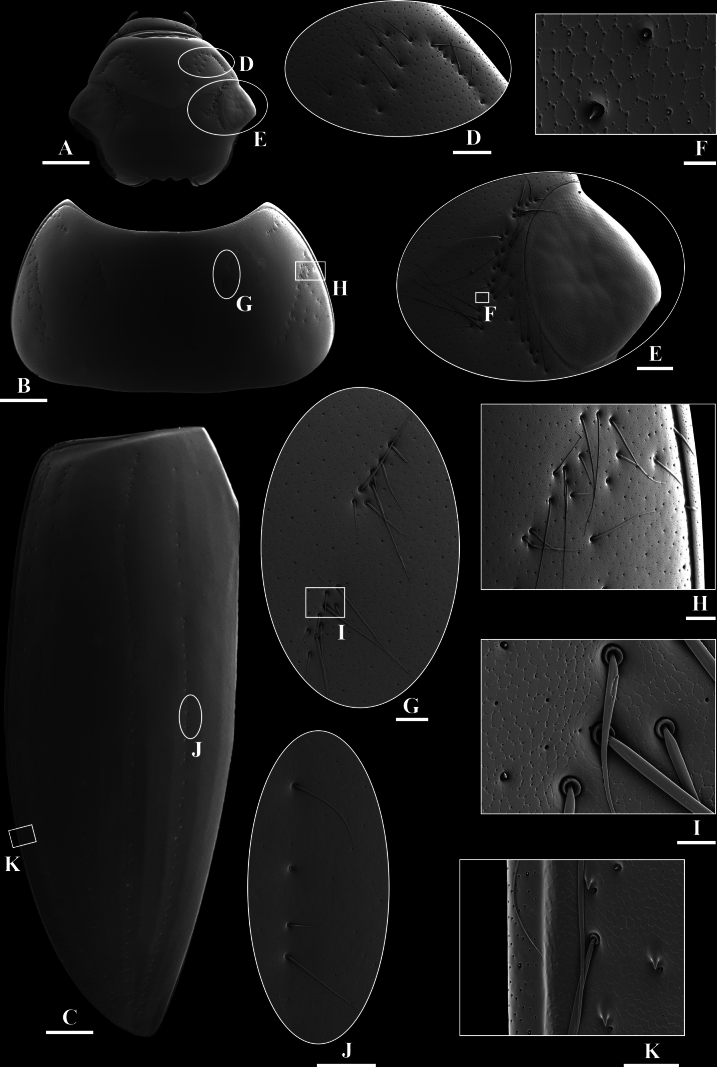
Scanning electron micrographs of *Hydrobiomorpha bachmanni* sp. nov. paratype (MACN-En 46708). **A**. Head, dorsal aspect; **B**. Pronotum, dorsal aspect; **C**. Elytron, dorsal aspect; **D–F**. Details of head; **G–I**. Details of pronotum; **J, K**. Details of elytron. Scale bars: 1 mm (**A–C**), 200 µm (**D, E, J**), 10 µm (**F**), 100 µm (**G–H**), 20 µm (**I**), 60 µm (**K**).

***Head*** (see Fig. 8A, D–F, paratype). Ground punctation double, with one type of punctures very fine, regular, and dense, the other fine, somewhat sparser and irregular; both well impressed, over very fine, regular microreticulation with a hexagonal pattern (Fig. 8D–F). Clypeus with anterior margin broadly emarginate, exposing a narrow membranous area; surface shining, without evident microreticulation. Four groups of coarse, deep punctures bearing trichobothria (systematic punctures, sensu Hansen 1991) on each side of clypeal midline and along inner margin of eyes (Fig. 8D, E). Labrum strongly transverse, anterolaterally rounded, with broad and shallow anteromedian emargination; surface shiny, without microreticulation, with fine double punctation. Two distinct pits, each bearing a tuft of trichobothria, present on either side of midline. Mentum with coarse, dense punctation. Submentum with sparse punctures restricted to lateral margins; central area smooth and shiny.

***Pronotum*** (see Fig. 8B, G–I, paratype). Transverse, arched, broadest at posterior margin, gradually narrowing toward anterior angles. Anterior margin arched; posterior margin very weakly bisinuate medially. Lateral margins narrowly reflexed. Anterior and posterior angles obtusely rounded. Surface shining, with very weakly impressed microreticulation of hexagonal pattern (Fig. 8I). Punctation double, less impressed than on head (Fig. 8G). Additionally, shallow coarse punctures bearing trichobothria present, on anterior angles, on lateral portions of disc (Fig. 8H), and along anterior third on each side of midline (Fig. 8G).

***Elytra*** (see Fig. 8C, J, K, paratype). Elongate-oval, broadest near mid-length. Subparallel in anterior 2/3, then uniformly rounded toward apex; weakly emarginate at suture. Anterior and lateral margins weakly reflexed, forming a narrow lateral bead (broadest at humerus and in anterior two-thirds, gradually narrowing toward apex), followed mesally by irregular field of moderate, rather dense punctures, extending from humerus to apex. Surface shining, with fine, shallow, isodiametric microreticulation of hexagonal pattern (Fig. 8J, K). Ground punctation composed of fine to very fine punctures, variable in size and density, not forming a clearly double punctation. Systematic punctures arranged in six rows, outer row intermingled with lateral puncture field.

***Venter*** (see Figs 2A, 4A). Prosternum with glabrous, slightly concave keel; posterior spine acute, moderately long, extending to mid-length of procoxae. Meso-metaventral keel with profile almost straight in lateral view, bearing small notch on anterior margin and depression along midline of metaventrite, area in which it appears slightly widened. Metaventral keel with posterior portion short, weakly arched ventrally, apex obtusely pointed, not reaching first abdominal ventrite. Fifth abdominal ventrite with glabrous area subtriangular, very broad, ~ 2/5 of its total length (Fig. 4A).

***Legs*** (see Figs 2A, 6A–F, paratype, 7A, G, paratype). Mesofemora with dense punctation, more impressed toward dorsal (anterior) margin; metafemora with very sparse and shallow punctation. Mesotibiae with setigerous furrow extending approximately along distal 1/3, gradually widening toward distal end and provided with dense brush of reddish setae (Fig. 7A, G). Protarsal claws robust, anterior (inner) claw bearing robust proximal tooth with pointed apex; meso- and metatarsal claws with conspicuous proximal tooth, though blunter (Fig. 6A–F).

***Genitalia*** (see Fig. 5A–D, paratype). Median lobe broad, apex deeply bifurcate into two long, narrow, subparallel projections. Dorsal sclerite markedly widened on distal third, gradually tapering proximally. Ventral membranous portion very narrow, distally bounded by more sclerotized bridge connecting both distal ends. Ventral sclerite long and narrow, clearly visible. Accessory appendages of median lobe broad, sclerotized, abruptly narrowed distally and terminating in short, apical hooks directed outward. Parameres moderately broad in lateral view, apex acute and strongly sclerotized, curved toward midline.

**Female**. Similar to male but slightly larger; differing in absence of mesotibial setigerous furrow. Glabrous area of fifth abdominal ventrite same length as in male but notably narrower (Fig. 4B). Claws falciform, relatively slender, with short distal portion; less robust and less strongly angulate than in male (Fig. 6G–L).

##### Etymology.

The species is named after Dr. Axel O. Bachmann (1927–2017), in recognition of his contributions to the taxonomy of South American Hydrophilidae and his lasting impact on the study of *Hydrobiomorpha*, including the description and revision of several Argentine species, as well as his role in building and curating reference collections that continue to support systematic research in the region.

##### Distribution.

Paraguay, Argentina: Corrientes Province.

##### Biology.

Unknown. This species was collected using light traps.

#### Hydrobiomorpha corumbaensis

Taxon classificationAnimaliaColeopteraHydrophilidae

Mouchamps, 1959

1B226020-0A1A-585D-9E64-9CFE39BDF1F9

Figs 1B, 2B, 3B, 4C, 4D, 5E–H, 6M, 7B

Hydrobiomorpha [(Hydrobiomorpha)] longa subsp. corumbaensis Mouchamps, 1959: 331 (original description).
Hydrobiomorpha corumbaensis : Contartese and Bachmann 1987: fig. 4 (distribution only).
Hydrobiomorpha corumbaensis : Bachmann 1988: 10, 21–22, fig. 8A–K (specific rank; redescription; key to species of the Americas).

##### Type locality.

Brazil: Mato Grosso, Corumbá [19°00'32"S, 57°39'10"W].

##### Material examined.

Argentina: • Corrientes Province: Cambyretá, 27°49'00"S, 56°50'00"W, 16.xi.2018, JI Urcola, G Rodriguez and SA Mazzucconi leg., light trap, 2 ♂♂ (LEBA); • Manantiales [29°21'26"S, 59°17'44"W], 9.x.1962, 1 ♂ (LEBA); • San Cosme [27°22'13"S, 58°30'45"W], 1937, J Wurth leg., 1 ♀ (MACN-En) and 1 ♂ (LEBA). • Entre Ríos Province: El Palmar National Park, small lagoon near Capilla Stream, 31°51'55"S, 58°19'04"W, 28.ii.2004, PLM Torres leg., 1 ♂ (LEBA); • La Paz [30°44'26"S, 59°38'40"W], xii.1972, 1 ♂ (LEBA); • Victoria [32°37'00"S, 60°10'00"W], 8–12.ii.1982, AO Bachmann leg., light trap, 1 ♂ (MACN-En).

##### Diagnosis.

L: 15.5–17.5 mm (males), 15.5–18.6 mm (females). W: 6.6–7.6 mm (males), 6.6–8.1 mm (females). Body shape (Figs 1B, 2B, 3B): navicular, tapered posteriorly and less so anteriorly, slightly convex; elytral margins not reflexed. Prosternal keel (Fig. 3B): ventral margin nearly straight; posterior spine acute, moderately long. Metaventral keel (Fig. 3B): posteriorly blunt and ventrally directed, not reaching first abdominal ventrite. Male: mesotibial setigerous furrow absent (Table 1; Fig. 7B); fifth abdominal ventrite with a short, poorly delimited glabrous area ~ 1/4 (in some cases ≤1/3) of its total length (Fig. 4C); protarsal claws dissimilar, with rounded proximal internal process; mesotarsal claws robust, with rounded proximal tooth; metatarsal claws very dissimilar, posterior claw with blunt, rounded preapical expansion (Fig. 6M); genitalia: median lobe ending in two very short projections; accessory appendages of median lobe broad in proximal half, becoming narrower distally and diverging outward, with rounded distal ends; parameres broad, abruptly narrowed distally (Fig. 5E–H). Female: glabrous area of fifth abdominal ventrite absent (Fig. 4D).

##### Distribution.

Brazil (Mouchamps 1959; Bachmann 1988), Paraguay (Bachmann 1988), Argentina (provinces): Corrientes (Bachmann 1988; Gómez Lutz et al. 2012; this study), Entre Ríos (Torres et al. 2007; this study).

##### Biology.

This species inhabits small permanent ponds (2,500–5,000 m^2^ in surface area) fully exposed to sunlight, with vegetated margins and centers, semi-transparent water, muddy substrate, and depth of < 2 m (Torres et al. 2007; Gómez Lutz et al. 2012).

##### Remarks.

It can be distinguished from other similar species by the unusual posterior metatarsal claw of males, which bears a blunt, rounded preapical expansion (Fig. 6M); in *H. longa* this expansion is angulate, rather than rounded (Fig. 6N). Male genitalia of *H. corumbaensis* (Fig. 5E–H) are similar to those of *H. longa* (Fig. 5Q–T) but differ in the median lobe, which ends in two short, blunt projections rather than long and sharp as in *H. longa*.

#### Hydrobiomorpha iguazu

Taxon classificationAnimaliaColeopteraHydrophilidae

Bachmann, 1988

67E4541A-C6BA-5F2F-BB44-4EB104463DE8

Figs 1C, 2C, 3C, 4E, 4F, 5I–L

Hydrobiomorpha iguazu : Contartese and Bachmann 1987: fig. 3 (nomen nudum, distribution only).
Hydrobiomorpha iguazu Bachmann, 1988: 9, 15–16, fig. 31A–H (original description; key to species of the Americas).
Hydrobiomorpha iguazu : Bachmann et al. 2002: 141 (female description).

##### Type locality.

Argentina: Misiones Province, Parque Nacional Iguazú [25°31'05"S, 54°08'00"W].

##### Material examined.

Argentina: • Misiones Province: Iguazú National Park [25°31'05"S, 54°08'00"W], x.1980, 1 ♂ (holotype, MACN-En 2213); • same locality iv.2001, PLM Torres and MC Michat leg., 1 ♀ (MACN-En).

##### Diagnosis.

L: 22.6 mm (male holotype), 21.0 mm (female). W: 10.6 mm (both sexes). Body shape (Figs 1C, 2C, 3C): subelliptical, slightly convex; elytral margin moderately reflexed on the posterior 2/3. Prosternal keel (Fig. 3C): moderately long, ventral margin nearly straight; posterior spine very short. Metaventral keel (Fig. 3C): slightly broadened, ending posteriorly in a short, laterally compressed, blunt tip, reaching approximately mid-length of first abdominal ventrite. Male: mesotibial setigerous furrow present, short, extending for ~ 1/4 of tibial length, with a short reddish setal brush (Table 1); fifth abdominal ventrite with a very large, subquadrate glabrous area, ~ 3/4 of its total length (Fig. 4E); protarsal claws unknown, not preserved in holotype (only known male); mesotarsal claws robust, with conspicuous proximal tooth; metatarsal claws robust, with conspicuous proximal tooth; genitalia: strongly sclerotized, median lobe subparallel, abruptly narrowed distally, apex bifurcate into two short sharp projections; accessory appendages of median lobe elongate, strongly sclerotized, with acute distal ends; parameres shorter than median lobe, broad in lateral view, narrowed distally and ending in projections, slightly curved towards midline (Fig. 5I–L). Female: fifth abdominal ventrite with a very large subtriangular glabrous area, ~ 2/3 of its total length (Fig. 4F).

##### Distribution.

Argentina: Misiones Province (Bachmann 1988; Bachmann et al. 2002).

##### Biology.

The female was collected from a shallow pond of turbid water (~ 20 cm deep), near the entrance to the Macuco Trail in Iguazú National Park. No habitat data are available for the holotype.

##### Remarks.

The large size, 21.0–22.6 mm and dark dorsal coloration resemble those of *H. utinga* Bachmann, 1988, *H. mucajai* Bachmann, 1988, *H. solitaria* Bachmann, 1988, and *H. praesumptapolita* Bachmann, 1988. The median lobe of the male genitalia resembles that of *H. praesumptapolita* (Bachmann, 1988). The large bare area on the last abdominal ventrite, and the short setigerous furrow, of 1/4 the length of the mesotibiae of males, are distinctive; the above mentioned species have the furrow longer, half as long as the tibiae, and in *H. mucajai* it is absent.

#### Hydrobiomorpha irina irina

Taxon classificationAnimaliaColeopteraHydrophilidae

(Brullé, 1837)

141480FD-9D83-5135-990A-4D0FAA1A3EA4

Figs 1D, 2D, 3D, 4G–H, 5M–P, 7C

Hydrophilus irinus Brullé, 1837: 55 (original description, see Bousquet (2016)).
Neohydrophilus irinus : Knisch 1924: 235.
Neohydrophilus medius : d'Orchymont 1939b: 263–264 (misidentification).
Hydrobiomorpha [(Hydrobiomorpha)] irina : Mouchamps 1959: 322, 326–327.
Hydrobiomorpha (Hydrobiomorpha) irina : Bachmann 1963: 30–31, fig. 1 (key to Argentine species).
Hydrobiomorpha irina irina : Contartese and Bachmann 1987: fig. 1 (distribution only).
Hydrobiomorpha irina irina : Bachmann 1988: 8, 26–28, fig. 12A–I (subspecific rank; redescription; key to species of the Americas).

##### Type locality.

[Argentina]: Rivière du Parana, “entre son embouchure et Corrientes”.

##### Material examined.

Argentina: • Buenos Aires Province: Verónica [35°23'00"S, 57°20'00"W], x.1937, JB Daguerre leg., 1 ♂ (MACN-En). • Chaco Province: Juan José Castelli [25°57'00"S, 60°37'00"W], 30.iii.1978, GJ Williner leg., 1 ♀ (MACN-En). • Corrientes Province: Cambyretá, 27°49'00"S, 56°50'00"W, 16.xi.2018, JI Urcola, G Rodriguez and SA Mazzucconi leg., light trap, 2 ♂♂ and 1 ♀ (LEBA); • Iberá Lagoon, 28°32'46"S, 57°11'45"W, 8.xi.2019, JI Urcola and G Rodriguez leg., light trap, 1 ♂ (LEBA); • Santo Tomé [28°33'00"S, 56°03'00"W] [no date], G Pellerano leg., 1 ♂ and 1 ♀ (MACN-En). • Entre Ríos Province: El Palmar National Park, small lagoon near the mouth of Capilla Stream, 31°51'55"S, 58°19'04"W, 28.ii.2004, PLM Torres leg., 2 ♂♂ and 1 ♀ (LEBA). • Santa Fe Province: El Cristal Natural Reserve, 30°01'05"S, 60°06'17"W, xii.2010, MC Michat leg., 1 ♀ (LEBA); • Rosario [32°57'27"S, 60°38'22"W], ii.1930, A Stevenin leg., 1 ♂ (MACN-En).

##### Diagnosis.

L: 14.2–16.0 mm (males), 14.5–17.2 mm (females). W: 6.4–7.3 mm (males), 6.5–8.0 mm (females). Body shape (Figs 1D, 2D, 3D): navicular, more or less attenuated anteriorly and posteriorly, slightly convex; elytral margin weakly reflexed or not reflexed. Prosternal keel (Fig. 3D): ventral margin nearly straight; posterior spine acute, moderately long. Metaventral keel (Fig. 3D): long and sharp, reaching or slightly surpassing posterior margin of first abdominal ventrite. Male: mesotibial setigerous furrow absent (Table 1; Fig. 7C); fifth abdominal ventrite with small glabrous area, < 1/4 of its total length (Fig. 4G); protarsal claws nearly straight, with long, slender proximal tooth; mesotarsal claws similar, with long, slender, slightly hooked proximal tooth; metatarsal claws slightly dissimilar, anterior claw with shorter, more strongly hooked proximal tooth; genitalia: median lobe broad proximally, narrowed at mid-length, and expanded distally, apex rounded, with two lateral projections directed proximally; accessory appendages of median lobe elongate, excurved, with acute distal ends; parameres shorter than median lobe, narrow and weakly curved ventrally (Fig. 5M–P). Female: fifth abdominal ventrite with a small glabrous area, ~ 1/5 or less of its total length (Fig. 4H).

##### Distribution.

Brazil (Mouchamps 1959; Bachmann 1963), Paraguay (Mouchamps 1959), Uruguay (Bachmann 1988), Argentina (provinces): Buenos Aires (Bachmann 1963, 1988; this study), Chaco (Mouchamps 1959; Bachmann 1963, 1988; Libonatti et al. 2013; this study), Corrientes (Bachmann 1963, 1988; Gómez Lutz et al. 2012; this study), Entre Ríos (Bachmann 1963, 1988; Torres et al. 2007), Santa Fe (Bachmann 1963, 1988; Macchia et al. 2015; this study), Tucumán (Bachmann 1988).

##### Biology.

The species was collected from a variety of lentic habitats, including temporary and semi-permanent ponds, shallow pools, and flooded areas. Habitats ranged from small, sun-exposed ponds (< 20 cm deep and ≤ 100 m^2^) with turbid water and muddy substrate (Libonatti et al. 2013; Torres et al. 2007) to larger, permanent water bodies (≤ 2 m deep and ≤ 2500 m^2^) (Torres et al. 2007; Gómez Lutz et al. 2012). Sites had variable turbidity (from semi-transparent to highly turbid water rich in organic matter), muddy substrates, and marginal and emergent aquatic vegetation including floating macrophytes. Some localities were temporary, with surface area and depth fluctuating due to desiccation, while others were more permanent. All collection sites were exposed to full sunlight and located in areas with low anthropogenic impact. Adults were also collected using light traps (Libonatti et al. 2013; Macchia et al. 2015; this study).

##### Remarks.

It can be distinguished from other similar species by the small glabrous area of the fifth abdominal ventrite (~ 1/4 of its total length in males (Fig. 4G), 1/5 in females (Fig. 4H)) and the absence of the male mesotibial setigerous furrow (Fig. 7C). *Hydrobiomorpha spinosa* is the most similar species, sharing a long metaventral keel that reaches or occasionally surpasses the posterior margin of the first abdominal ventrite; however, *H. spinosa* has a very long male mesotibial furrow (~ 2/3 of tibial length (Fig. 7F, J)) and a minute or absent glabrous area on the fifth abdominal ventrite (Fig. 4M). *Hydrobiomorpha irina sesquirina*, *H. irina varginha*, and *H. irinoides* (all Bachmann 1988) are larger; in *H. irina sesquirina* the fifth abdominal ventrite is slightly tectiform, with the glabrous area extending anteriorly as a narrow strip.

#### Hydrobiomorpha longa

Taxon classificationAnimaliaColeopteraHydrophilidae

(Bruch, 1915)

B590A4CC-7FEB-57D9-A39A-4F93E1CD0496

Figs 1E, 2E, 3E, 4I–J, 5Q–T, 6N, 7D, 7H

Hydrophilus longus Bruch, 1915: 448–449, fig. 1 (original description).
Neohydrophilus longus : d’Orchymont 1919: 161–162.
Hydrobiomorpha [(Hydrobiomorpha)] longa : Mouchamps 1959: 327, 330–331.
Hydrobiomorpha (Hydrobiomorpha) longa : Bachmann 1963: 30–32, fig. 2 (key to Argentine species).
Hydrobiomorpha longa : Contartese and Bachmann 1987: fig. 5 (distribution only).
Hydrobiomorpha longa : Bachmann 1988: 10, 19–21, fig. 7A–H (redescription; key to species of the Americas).

##### Type locality.

Argentina: Santa Fe Province, La Gallareta [29°34'00"S, 60°23'00"W].

##### Material examined.

Argentina: • Buenos Aires Province: Zárate, camping Isla Talavera, 33°54'35"S, 58°56'12"W, 15.xi.2025, G Rodriguez leg., light trap, 1 ♂ (LEBA). • Chaco Province: Presidencia Roque Sáenz Peña [26°47'00"S, 60°27'00"W], 30.xi.1980, 2 ♂♂ (MACN-EN). • Entre Ríos Province: Concordia [32°23'50"S, 58°01'02"W] [no date], JB Daguerre leg., 1 ♂ (LEBA); • La Paz [30°44'26"S, 59°38'40"W], xii.1974, 1 ♂ (LEBA); • Pronunciamiento [32°21'00"S, 58°26'00"W], ii.1958, L Gontero leg., 1 ♀ (MACN-En); • Liebig [32°09'12"S, 58°11'28"W], x.1988, MR Zelich leg., light trap, 2 ♂♂ (LEBA); • Victoria [32°37'00"S, 60°10'00"W], 21–26.iii.1981, AO Bachmann leg., 2 ♂♂ and 5 ♀♀ (LEBA); • Villa Elisa [32°09'47"S, 58°24'02"W], xi.1973, L Gontero leg., 2 ♂♂ (LEBA). • Santa Fe Province: Rosario [32°57'27"S, 60°38'22"W], ii.1930, A Stevenin leg., 1 ♂ (MACN-En).

##### Diagnosis.

L: 14.0–15.3 mm (males), 14.8–16.6 mm (females). W: 6.2–6.8 mm (males), 6.9–7.5 mm (females). Body shape (Figs 1E, 2E, 3E): narrow, sides subparallel at mid-length, slightly tapered posteriorly and anteriorly, slightly convex; elytral margins not reflexed. Prosternal keel (Fig. 3E): moderately long, ventral margin nearly straight; posterior spine short, acute. Metaventral keel (Fig. 3E): moderately developed, posterior tip short and sharp, not reaching first abdominal ventrite. Male: mesotibial setigerous furrow absent (Table 1; Fig. 7D, H); ventral inner face of mesotibia with only a small distal group of spines; fifth abdominal ventrite with glabrous area ~ 1/3 of its total length (Fig. 4I); protarsal claws with robust proximal tooth; mesotarsal claws with rounded, broad proximal tooth; metatarsal claws with posterior claw bearing conspicuous angular preapical expansion (Fig. 6N); genitalia: median lobe slender, gradually tapering distally; apex bifurcate with two long sharp projections directed distally; accessory appendages of median lobe elongate, sclerotized, divergent distally, with truncate distal ends; parameres shorter than median lobe, narrow in lateral view, gradually narrowed distally and ending in rather blunt projections directed dorsally (Fig. 5Q–T). Female: fifth abdominal ventrite without glabrous area (Fig. 4J).

##### Distribution.

Paraguay (Mouchamps 1959; Bachmann 1963, 1988), Argentina (provinces): Buenos Aires (Bachmann 1963, 1988; this study), Chaco (Bachmann 1988; Libonatti et al. 2013), Corrientes (Bachmann 1963, 1988), Entre Ríos (Bachmann 1963, 1988), Formosa (Bachmann 1963, 1988), Salta (Bachmann 1963), Santa Fe (Bruch 1915; d’Orchymont 1928; Mouchamps 1959; Bachmann 1963, 1988), Tucumán (Bachmann 1988).

##### Biology.

Adults inhabit moderately vegetated permanent ponds (this study) and have been recorded from a stream with unshaded, muddy substrate, relatively clear water, and floating vegetation (Libonatti et al. 2013), representing the only lotic habitat record for the genus in Argentina. Adults occasionally fly at night and are attracted to artificial lights (this study).

##### Remarks.

It can be distinguished from other similar species by the posterior metatarsal claw of males, which bears a conspicuous angular preapical expansion (Fig. 6N), and by being the smallest and narrowest species in the genus (14.0–16.6 mm). The largest specimens of *H. longa* resemble externally the smallest ones of *H. irina irina*; however, *H. irina irina* has a longer and sharper metaventral keel and a denser, more regular marginal series of elytral punctures. *H. corumbaensis*, originally described as a subspecies of *H. longa*, is larger and has a blunt metaventral keel and the posterior metatarsal claw of males bears a rounded rather than angular preapical expansion (Fig. 6M). The median lobe of the male genitalia ends in two long sharp projections in *H. longa* (Fig. 5Q, R) whereas in *H. corumbaensis* the projections are short and blunt (Fig. 5E, F).

#### Hydrobiomorpha rudesculpta

Taxon classificationAnimaliaColeopteraHydrophilidae

(d’Orchymont, 1939)

7B5F145C-EA0E-57C1-97F8-7608D3CA7724

Figs 1F, 2F, 3F, 4K, 4L, 5U–X, 6O, 7E, 7I

Neohydrophilus rudesculptus d’Orchymont, 1939a: 376–378 (original description).
Hydrobiomorpha rudesculpta : Mouchamps 1959: 324, 333.
Hydrobiomorpha (Hydrobiomorpha) rudesculpta : Bachmann 1963: 29, 32, fig. 3 (key to Argentine species).
[Neohydrophilus] rudesculptus: Döbler 1977: 386.
Hydrobiomorpha rudesculpta : Contartese and Bachmann 1987: fig. 2 (distribution only).
Hydrobiomorpha rudesculpta : Bachmann 1988: 8, 45–46, fig. 30A–H (redescription; key to species of the Americas).

##### Type locality.

Paraguay: Río Pilcomayo.

##### Material examined.

Argentina: • Chaco Province: Resistencia [27°27'05"S, 58°59'12"W] [no date], JB Daguerre leg., 1 ♂ (LEBA); • San Bernardo [27°17'30"S, 60°42'30"W], ii.1980, OR Di Iorio leg., light trap, 1 ♂ and 2 ♀♀ (LEBA). • Corrientes Province: Iberá lagoon, 28°32'46"S, 57°11'45"W, 7.xi.2019, JI Urcola and G Rodriguez leg., light trap, 1 ♀ (LEBA); • Manantiales [29°21'26"S, 59°17'44"W], ix.1960, 1 ♀ (LEBA). • Salta Province: Piquete [24°45'17"S, 64°28'08"W], 18.i.1928, A Bridarolli leg., 1 ♀ (MACN-En). • Santa Fe Province: Chaco [likely refers to the biogeographic province] [no date], C Bruch leg., 1 ♂ (MACN-En). • No locality data: [no date], A Breyer leg., 1 ♀ (MACN-En).

##### Diagnosis.

L: 16.3–18.8 mm (males), 17.2–20.3 mm (females). W: 8.8–9.9 mm (males), 9.4–11.6 mm (females). Body shape (Figs 1F, 2F, 3F): broadly oval, widest slightly posterior to mid-length of elytra, strongly convex (subglobose); elytral margins not reflexed. Prosternal keel (Fig. 3F): strongly oblique, posterior spine very long. Metaventral keel (Fig. 3F): very short, posterior tip blunt, not reaching mid-length of first abdominal ventrite. Male: mesotibial setigerous furrow absent (Table 1; Fig. 7E, I); ventral inner face of mesotibia with a very dense distal patch of spinules; claws highly distinctive; protarsal claws with enormous spatulate proximal tooth on inner claw; mesotarsal claws with very large, rounded, oblique proximal tooth; metatarsal claws long, with large, rounded proximal tooth (Fig. 6O); fifth abdominal ventrite with small, wide, poorly delimited glabrous area (Fig. 4K); genitalia: median lobe very broad, largely membranous, apex with two large rounded lobes; accessory appendages of median lobe narrow in dorsal view, broad in lateral view, strongly sclerotized, distally bent at a right angle toward the midline, bearing an acute outwardly directed process at the point of inflection; parameres slightly shorter than median lobe, with a subdistal dorsal projection directed proximally and a complex basal lobe (Fig. 5U–X). Female: fifth abdominal ventrite without glabrous area (Fig. 4L).

##### Distribution.

Paraguay (d’Orchymont 1939a; Mouchamps 1959; Bachmann 1963, 1988), Argentina (provinces): Chaco (Bachmann 1963, 1988; this study), Corrientes (Bachmann 1963, 1988; Torres et al. 2012; this study), Entre Ríos (Bachmann 1963, 1988), Formosa (Bachmann 1963, 1988), Salta (Bachmann 1963, 1988), Santa Fe (Bachmann 1963).

##### Biology.

To date, the biology of this species remains unknown; however, it is frequently collected using light traps (Torres et al. 2012; this study).

##### Remarks.

*Hydrobiomorpha rudesculpta* is the most anatomically distinctive Argentine species of the genus, recognizable by its subglobose body shape, the spatulate proximal tooth of the anterior protarsal claw in males (Fig. 6O), and the distinctive structure of the male genitalia (Fig. 5U–X; see Diagnosis for details). No other species combines these characters.

#### Hydrobiomorpha spinosa

Taxon classificationAnimaliaColeopteraHydrophilidae

(d’Orchymont, 1928)

AFB0D82B-5B98-5A62-84CA-5A2BF54D87B6

Figs 1G, 2G, 3G, 4M, 4N, 5Y–AB, 7F, 7J

Hydrophilus medius : Brullé, 1837: 54–55 (partim, only the record from Rio Negro, Patagonia).
Hydrochares medius : Berg 1884: C [= 100] (fide Bachmann 1988).
Neohydrophilus medius var. spinosus d’Orchymont, 1928: 164 (original description).
Hydrobiomorpha [(Hydrobiomorpha)] spinosa : Mouchamps 1959: 322, 327, 328.
Hydrobiomorpha spinosa : Bachmann 1963: 30, 32, 33, fig. 4 (key to Argentine species).
Hydrobiomorpha spinosa : Contartese and Bachmann 1987: fig. 4 (distribution only).
Hydrobiomorpha spinosa : Bachmann 1988: 8, 37–39, fig. 23A–I (redescription; key to species of the Americas).
Hydrobiomorpha spinosa : Archangelsky et al. 2004: 253–264 (description of immature stages).

##### Type localities.

Argentina: Santiago del Estero Province, bords du Rio Salado, environs d’Icaño, Mistol Paso [28°40'41"S, 62°53'02"W], and Chaco de Santiago del Estero, Rio Salado; Santa Fe Province, Chaco de Santa Fe, Las Garzas [28°50'46"S, 59°30'01"W], bords du Rio Las Garzas, 20 km à l’Ouest d’Ocampo.

##### Material examined.

Argentina: • Buenos Aires Province: Olavarría, Tapalqué stream, 36°53'36"S, 60°19'50"W, 23.i.2026, TA Favier Dubois leg., 3 ♀♀ (LEBA). • Chaco Province: Chaco National Park [26°48'31"S, 59°36'24"W],18.i.2011, MC Michat and SA Mazzucconi leg, 2 ♂♂ and 2 ♀♀ (LEBA); • Presidencia Roque Sáenz Peña [26°47'00"S, 60°27'00"W], 22.xii.1991, light trap, 1 ♂ (LEBA). • Entre Ríos Province: El Palmar National Park, 31°52'10"S, 58°13'39"W, 11.xi.2023, G Rodriguez leg., 1 ♀ (LEBA); • Victoria [32°37'00"S, 60°10'00"W], 23–26.iii.1981, AO Bachmann leg., 1 ♂ and 1 ♀ (LEBA). • Salta Province: Rosario de la Frontera, Camping ACA [25°50'03"S, 64°56'35"W], 27.xi.2003, light trap, PLM Torres and MC Michat leg., 1 ♂ and 4 ♀♀ (LEBA).

##### Diagnosis.

L: 13.7–17.9 mm (males), 14.0–20.5 mm (females). W: 6.6–8.3 mm (males), 6.7–10.3 mm (females). Body shape (Figs 1G, 2G, 3G): variable, ranging from elliptical to navicular, anterior and posterior ends tapered to different degrees, moderately convex; elytral margins not reflexed. Prosternal keel (Fig. 3G): variable, generally long; posterior spine either very long and robust or short and acute. Metaventral keel (Fig. 3G): very long, sharp, often slightly hooked, reaching or surpassing posterior margin of first abdominal ventrite. Male: mesotibial setigerous furrow present, very long, ~ 1/2–2/3 of tibial length, with dense reddish setal brush (Table 1; Fig. 7F, J); fifth abdominal ventrite with minute triangular glabrous area, commonly < 1/5 of its total length (Fig. 4M); protarsal claws slightly dissimilar, proximal tooth long, slender, somewhat flattened; mesotarsal claws robust, proximal tooth long, blunt; metatarsal claws slightly dissimilar, proximal tooth long, blunt; genitalia: median lobe narrow, subparallel; apex truncate, with two short rounded lateral projections directed proximally, visible in ventral view; dorsal sclerite as a prominent lamina projecting perpendicularly to the longitudinal axis of the median lobe; accessory appendages of median lobe elongate, excurved, slightly sigmoid, with acute distal ends; parameres shorter than median lobe, moderately narrow in lateral view, strongly curved ventrally (Fig. 5Y–AB). Female: fifth abdominal ventrite without glabrous area (Fig. 4N).

##### Distribution.

Brazil (d’Orchymont 1928), Paraguay (Mouchamps 1959; Bachmann 1963), Uruguay (Bachmann 1963; Benetti and Garrido 2004), Argentina (provinces): Buenos Aires (d’Orchymont 1928; Mouchamps 1959; Bachmann 1963, 1988; von Ellenrieder and Fernández 2000; Archangelsky et al. 2004; Fontanarrosa et al. 2004; Fernández et al. 2010; Macchia 2022; Macchia and Cicchino 2023; this study), Chaco (Bachmann 1963, 1988; Libonatti et al. 2013; this study), Chubut (Bachmann 1963), Córdoba (Bachmann 1963, 1988), Corrientes (Bachmann 1963, 1988; Torres et al. 2012), Entre Ríos (Bachmann 1963, 1988; Torres et al. 2007; this study), Formosa (Bachmann 1963, 1988), La Pampa (Bachmann 1988), Mendoza (Bachmann 1963, 1988), Misiones (Bachmann 1963, 1988), Río Negro (Bachmann 1988), Salta (Bachmann 1963, 1988; this study), San Juan (Bachmann 1988), San Luis (Bachmann 1988), Santa Fe (d’Orchymont 1928; Mouchamps 1959; Bachmann 1963, 1988), Santiago del Estero (Mouchamps 1959; Bachmann 1963, 1988), Tucumán (Bachmann 1963, 1988).

##### Biology.

*Hydrobiomorpha spinosa* is the most widely distributed American species of the genus. Adults are scavengers or periphyton eaters, inhabiting vegetated ponds, characterized by abundant organic matter, turbid water, and a muddy substrate. Adults occasionally fly at night and are attracted to artificial lights (Torres et al. 2007, 2012; Libonatti et al. 2013; Macchia and Cicchino 2023). Larvae are active predators but are rarely collected. Archangelsky et al. (2004) provided the first description of the egg-case for the genus and described all larval instars and the pupa.

##### Remarks.

It can be distinguished from other similar species by the very long male mesotibial setigerous furrow (1/2–2/3 of tibial length) and the minute or absent glabrous area of the fifth abdominal ventrite. Small specimens resemble *H. irina irina*; however, *H. irina irina* has a shorter metaventral keel, lacks the male mesotibial furrow, and has a well-developed glabrous area in both sexes (~ 1/4 of its total length in males, 1/5 in females). In *H. spinosa*, the glabrous area typically occupies about 1/5 of the total length of the fifth abdominal ventrite but may be reduced to ~ 1/10 (Bachmann 1988). The median lobe of the male genitalia of *H. irina irina* is broader, with a rounded apex bearing lateral projections (Fig. 5M, N), whereas in *H. spinosa* the apex is subtruncate with rounded lateral expansions (Fig. 5Y, Z).

#### Hydrobiomorpha utiariti

Taxon classificationAnimaliaColeopteraHydrophilidae

Bachmann, 1988

A34ABB4E-3A36-5C0C-A802-A7F2C61C2073

Figs 5AC–AF, 9

Hydrobiomorpha utiariti Bachmann, 1988: 8, 18, 19, fig. 6A–K (original description).

##### Type locality.

Brazil: Mato Grosso Norte, Utiariti, Río Papagaio [13°01'38"S, 58°16'57"W].

##### Material examined.

Brazil: • Mato Grosso, Utiariti, Río Papagaio [13°01'38"S, 58°16'57"W], xi.1966, JM Viana leg., 1 ♂ (holotype, MACN-En 2746) and 1 ♀ (allotype of Bachmann 1988, MACN-En 2747). Paraguay: • Puerto Vallemí [22°09'27"S, 57°57'05"W], 11.vi.1952, AO Bachmann leg., 1 ♀ (paratype, MACN-En 2748).

##### Diagnosis.

**Figure 9.**
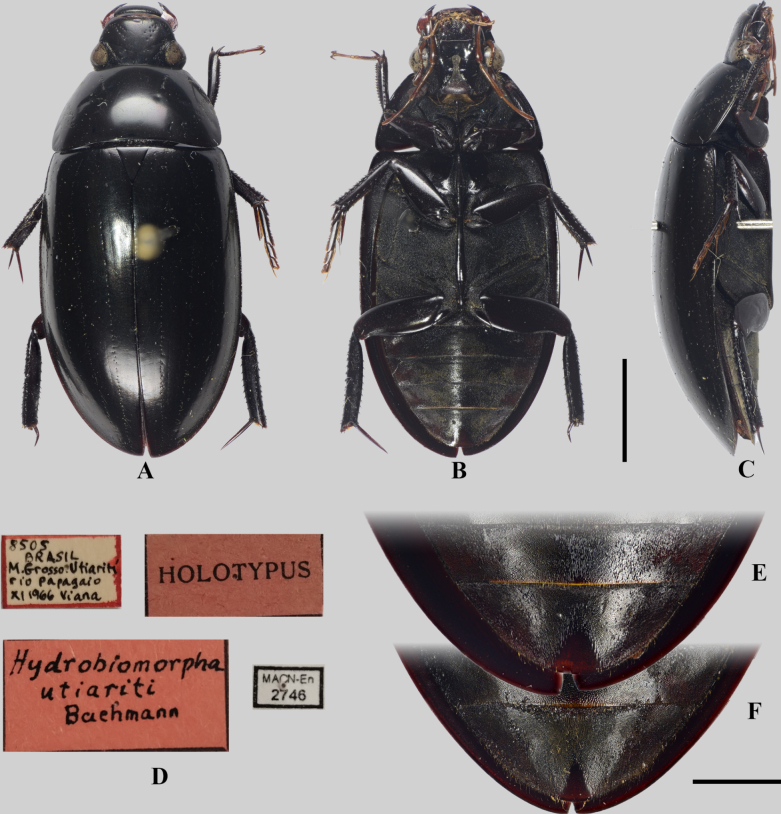
**A–C**. Habitus of *Hydrobiomorpha utiariti* holotype (MACN-En 2746); **A**. Dorsal aspect; **B**. Ventral aspect; **C**. Lateral aspect; **D**. Labels of the *Hydrobiomorpha utiariti* holotype; **E**. Abdominal ventrites IV–V of *Hydrobiomorpha utiariti* holotype, ventral aspect; **F**. Abdominal ventrites IV–V of *Hydrobiomorpha utiariti* female labelled as allotype (MACN-En 2747), ventral aspect. Scale bars: 5 mm (**A–C**), 2 mm (**E, F**).

L: 22.0 mm (male holotype), 21.0–22.5 mm (females). W: 10.0 mm (male holotype), 9.4–10.0 mm (females). Body shape (Fig. 9A, B): navicular, sides subparallel, slightly expanded at mid-length, moderately convex; elytral margins slightly reflexed, especially posteriorly, more distinctly in males than in females. Prosternal keel (Fig. 9C): ventral margin straight, posterior spine very short. Metaventral keel (Fig. 9C): slightly broadened, with a fine longitudinal groove; posterior end rounded, reaching middle of first abdominal ventrite. Male: mesotibial setigerous furrow present, ~ 1/2 of tibial length, narrow, with a dense brush of reddish setae (Table 1); fifth abdominal ventrite with a subtriangular, very large glabrous area, ~ 2/3 of its total length (Fig. 9E); claws robust; protarsal claws with robust proximal tooth on inner claw; mesotarsal claws with somewhat spatulate proximal tooth; metatarsal claws unknown, not preserved in the holotype (only known male); genitalia: median lobe broad, apex truncate, bearing two slightly divergent projections arising centrally from the margin; accessory appendages of median lobe elongate, strongly sclerotized, abruptly narrowed distally, terminating in short outwardly directed apical hooks; parameres as long as the median lobe, broad in lateral view, strongly curved, with subdistal denticulations and apices directed toward the midline (Fig. 5AC–AF). Female: fifth abdominal ventrite with subtriangular glabrous area, < 1/2 of its total length (Fig. 9F).

##### Distribution.

Brazil (Bachmann 1988), Paraguay (Bachmann 1988).

##### Biology.

Unknown.

##### Remarks.

*Hydrobiomorpha utiariti* is included herein because it overlaps in distribution with *H. bachmanni* sp. nov. in Paraguay and presents similar anatomical features, particularly in body size, convexity, and development of male mesotibial setigerous furrow and glabrous area of fifth abdominal ventrite, which may lead to confusion between the two species. *Hydrobiomorpha utiariti* differs from *H. bachmanni* sp. nov. by its larger size, darker coloration, longer male mesotibial setigerous furrow (~ 1/2 of tibial length, whereas in *H. bachmanni* sp. nov. it extends ~ 1/3), and larger glabrous area in both sexes (~ 2/3 of its total length in males (Fig. 9E) and < 1/2 in females (Fig. 9F), whereas ~ 2/5 in both sexes in *H. bachmanni* sp. nov. (Fig. 4A, B). It also differs in the prosternal keel, which is shorter in *H. utiariti* and bears a short posterior spine, and in the shape of the male genitalia: the median lobe has two slightly divergent apical projections in *H. utiariti* (Fig. 5AC–AF), whereas in *H. bachmanni* sp. nov. these are subparallel (Fig. 5A–D); and the parameres of *H. utiariti* bear conspicuous subdistal internal denticulations that are absent in *H. bachmanni* sp. nov.

### Checklist of Argentine *Hydrobiomorpha*

1. *Hydrobiomorpha bachmanni* sp. nov.: Argentina, Paraguay.
2. *Hydrobiomorpha corumbaensis* Mouchamps, 1959: Argentina, Brazil, Paraguay.
3. *Hydrobiomorpha iguazu* Bachmann, 1988: Argentina.
4. *Hydrobiomorpha irina irina* (Brullé, 1837): Argentina, Brazil, Paraguay, Uruguay.
5. *Hydrobiomorpha longa* (Bruch, 1915): Argentina, Paraguay.
6. *Hydrobiomorpha rudesculpta* (d’Orchymont, 1939): Argentina, Paraguay.
7. *Hydrobiomorpha spinosa* (d'Orchymont, 1928): Argentina, Brazil, Paraguay, Uruguay.

#### Key to Argentine *Hydrobiomorpha*

**Table:** 

1	Body broadly oval, strongly convex, subglobose in lateral view (Figs 1F, 2F, 3F); prosternal keel strongly oblique, with a very long posterior spine (Fig. 3F); male anterior protarsal claw with large spatulate proximal tooth (Fig. 6O)	***H. rudesculpta* (d’Orchymont)**
–	Body elliptical to navicular, or narrow with subparallel sides, weakly convex in lateral view, never subglobose; prosternal keel slightly concave or with ventral margin nearly straight, not strongly oblique, posterior spine short to moderately long; male anterior protarsal claw without large spatulate proximal tooth	**2**
2	Metaventral keel very long, reaching or surpassing the posterior margin of the first abdominal ventrite (Fig. 3D, G)	**3**
–	Metaventral keel shorter, not reaching the posterior margin of the first abdominal ventrite (Fig. 3A–C, E)	**4**
3	Body shape navicular, weakly convex (Figs 1D, 2D, 3D); male mesotibial setigerous furrow absent (Fig. 7C); fifth abdominal ventrite with small but distinct glabrous area in both sexes (Fig. 4G, H)	***H. irina irina* (Brullé)**
–	Body shape elliptical to navicular, moderately convex (Figs 1G, 2G, 3G); male mesotibial setigerous furrow present, very long, almost 2/3 of tibial length (Fig. 7F, J); fifth abdominal ventrite with minute or absent glabrous area in males, absent in females (Fig. 4M, N)	***H. spinosa* (d’Orchymont)**
4	Body large, > 21 mm; glabrous area of fifth abdominal ventrite very large in both sexes (ca. 3/4 of its total length in males, ca. 2/3 in females); elytral margins moderately reflexed on the posterior two-thirds (Figs 1C, 2C, 3C);	***H. iguazu* Bachmann**
–	Body < 21 mm; glabrous area of fifth abdominal ventrite, when present, smaller, occupying < 1/2 of its total length; elytral margin not reflexed or weakly reflexed	**5**
5	Males with mesotibial setigerous furrow present, ~ 1/3 of tibial length (Fig. 7A, G); glabrous area of fifth abdominal ventrite present in both sexes, ~ 2/5 of its total length (Fig. 4A, B)	***H. bachmanni* sp. nov**.
–	Males with mesotibial setigerous furrow absent; glabrous area of fifth abdominal ventrite absent in females	**6**
6	Metaventral keel with posterior tip short and sharp (Fig. 3E); males with posterior (ventral) metatarsal claw bearing a conspicuous angular preapical expansion (Fig. 6N); median lobe of male genitalia emarginate apically, with two long, sharp lateral projections (Fig. 5Q–T)	***H. longa* (Bruch)**
–	Metaventral keel posteriorly blunt and ventrally directed (Fig. 3B); males with posterior (ventral) metatarsal claw bearing a blunt, rounded preapical expansion (Fig. 6M); median lobe of male genitalia emarginate apically, with two short, blunt lateral projections (Fig. 5E–H)	***H. corumbaensis* Mouchamps**

## Discussion

The most informative characters identified for species recognition in Argentine *Hydrobiomorpha* include the male mesotibial setigerous furrow, the glabrous area of the fifth abdominal ventrite, claw anatomy, and male genitalia. The mesotibial furrow shows consistent interspecific variation in length and position and appears to be relatively stable within species, although some variability was observed in *H. spinosa*. This character was recognized by Bachmann (1988) but not illustrated, and despite its apparent diagnostic value it has not been incorporated into subsequent regional revisions, including those focused on Australia (Watts 1990), Central America (Short 2004), the Afrotropical region (Bilton 2016), and India (Sonali et al. 2025). The glabrous area of the fifth abdominal ventrite proved to be another reliable diagnostic character, particularly regarding its relative size, shape, and occurrence in both sexes. Claw anatomy also provides useful diagnostic features in some species, especially the spatulate proximal tooth of the anterior protarsal claw in *H. rudesculpta* and the shape of the posterior metatarsal claw in *H. longa* and *H. corumbaensis*. Male genitalia remain essential for species identification, particularly among externally similar taxa, where features of the median lobe and parameres provides consistent species-level differences. Although generally stable within species, no single character alone was sufficient for the identification of all taxa, reinforcing the importance of combining external anatomy with male genitalia in species identification within the genus.

This study increases the number of recognized Argentine species of *Hydrobiomorpha* from six to seven, representing an increase in the known diversity of the genus in the country after nearly four decades without new species descriptions. The description of *H. bachmanni* sp. nov. based on material from northeastern Argentina and Paraguay highlights that even in Neotropical groups considered relatively well known, undocumented diversity persists.

Distribution patterns among Argentine species vary markedly in extent and apparent range size. *Hydrobiomorpha spinosa* has the broadest recorded distribution, occurring in northern and central Argentina and extending into neighboring countries such as Brazil, Paraguay, and Uruguay. In contrast, *H. iguazu* is currently known only from Misiones Province, associated with the Paranaense Forest ecoregion. However, this apparent restriction may reflect limited sampling, particularly in the Paranaense Forest of Paraguay and southern Brazil. In addition, extensive habitat fragmentation and loss in this region may further complicate the interpretation of current distributional patterns. Distributional data presented here are based primarily on museum collections and targeted collecting efforts, which may not fully capture the geographic ranges of individual species. Systematic sampling across Argentina’s diverse ecoregions, particularly in under-collected areas such as the Dry Chaco, Yungas, and Patagonian steppe, would likely yield new records and potentially reveal additional undescribed species.

Species richness is highest in northeastern Argentina, with all seven species collectively recorded from Entre Ríos, Corrientes, and Misiones provinces. This pattern may be associated with the absence of major biogeographic barriers and the presence of fluvial corridors (Paraná, Uruguay, and Paraguay rivers), which likely facilitate species dispersal in this region, corresponding to the southern transition of the Paranaense Province (Arana et al. 2021). However, northeastern Argentina is also one of the best sampled regions for aquatic Coleoptera, as evidenced by numerous studies conducted in the area (e.g.: Torres et al. 2007, 2012; Fernández et al. 2008; Gómez Lutz et al. 2012; Libonatti et al. 2013; Macchia et al. 2015; Urcola et al. 2025), and the observed richness pattern may partly reflect sampling bias rather than a true biogeographic signal.

Ecological data for the genus remain fragmentary worldwide. Available records indicate that most species inhabit vegetated permanent ponds with muddy substrates, although *H. longa* has also been collected from a stream with floating vegetation. The lack of ecological information for *H. bachmanni* sp. nov., *H. rudesculpta*, and *H. utiariti* highlights the need for targeted field studies documenting habitat preferences, seasonal activity, and larval ecology. These data are essential for understanding the functional roles of these beetles in freshwater ecosystems and for assessing potential conservation concerns.

Despite the progress made, several limitations should be acknowledged. The phylogenetic relationships among Argentine species and their placement within the broader Neotropical fauna remain unresolved. Molecular phylogenetic analyses would provide a framework for testing species group monophyly and understanding the evolutionary history of the genus in South America. Additionally, the immature stages of most Argentine species remain unknown. While *H. spinosa* has been described in detail (Archangelsky et al. 2004), larvae of other species have yet to be associated with adults. Future rearing efforts and/or molecular association techniques will be necessary to document larval anatomy and asses its potential value for species delimitation. Finally, the taxonomic status of some South American *Hydrobiomorpha* species, particularly those described from single localities or based on limited material, merits re-examination with modern approaches.

High-resolution digital imaging and scanning electron microscopy proved useful for documenting subtle anatomical variation such as that of the prosternal and metaventral keels, the mesotibial setigerous furrow, and male genitalia. These tools complement traditional line drawings and facilitate interpretation of diagnostic characters, particularly in anatomically similar species.

This revision updates the taxonomy of Argentine *Hydrobiomorpha* based on high-resolution imaging and re-evaluation of anatomical features. The description of *H. bachmanni* sp. nov. adds to the known diversity of the genus in the region. Continued exploration of poorly sampled areas, combined with molecular and ecological approaches, is likely to reveal further species and improve our understanding of the distribution and biology of this group in southern South America.

## Supplementary Material

XML Treatment for Hydrobiomorpha

XML Treatment for Hydrobiomorpha bachmanni

XML Treatment for Hydrobiomorpha corumbaensis

XML Treatment for Hydrobiomorpha iguazu

XML Treatment for Hydrobiomorpha irina irina

XML Treatment for Hydrobiomorpha longa

XML Treatment for Hydrobiomorpha rudesculpta

XML Treatment for Hydrobiomorpha spinosa

XML Treatment for Hydrobiomorpha utiariti

## Additional information

### Artificial Intelligence (AI) use

The authors accept full responsibility for the content of the manuscript, including the disclosure of any use of AI.

No AI tools were used in the preparation of this manuscript.

### Author ORCIDs

M.R. Urcola https://orcid.org/0000-0002-1335-0072

G. Rodriguez https://orcid.org/0000-0002-0201-2932

M.C. Michat https://orcid.org/0000-0002-1962-7976

P.L.M. Torres https://orcid.org/0000-0002-6532-9347

### Data availability

All of the data that support the findings of this study are available in the main text.
